# Supplementary material for: Causal Relationship between Aging and Anorexia Nervosa: A White-Matter-Microstructure-Mediated Mendelian Randomization Analysis
Source: Biomedicines. 2024 Aug 16;12(8):1874. doi: 10.3390/biomedicines12081874 (PMC11351342; doi:10.3390/biomedicines12081874)
Supplement: Supplementary file 1 [file biomedicines-12-01874-s001.zip › Supplementary Figure/Figure S4/Singe SNP plot of MR analysis for TL to ubm-b-2123.pdf]

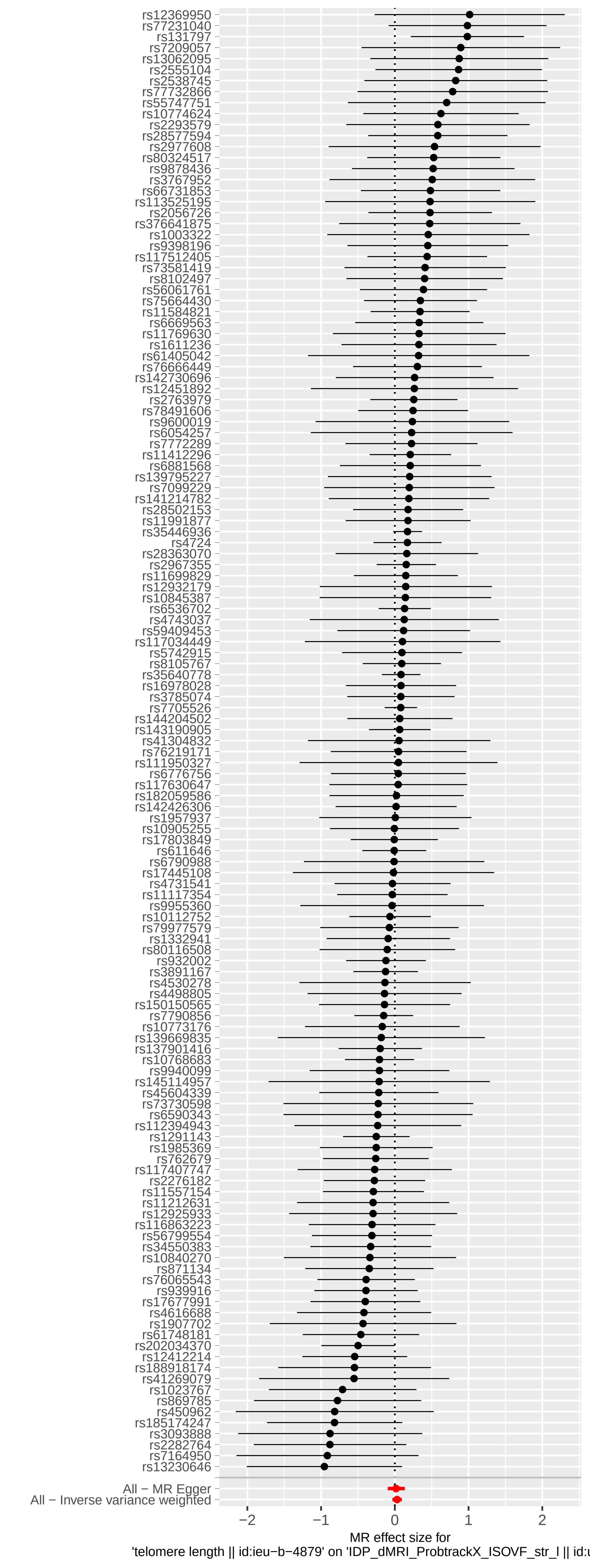

rs12369950
rs77231040
rs131797
rs7209057
rs13062095
rs2555104
rs2538745
rs77732866
rs55747751
rs10774624
rs2293579
rs28577594
rs2977608
rs80324517
rs9878436
rs3767952
rs66731853
rs113525195
rs2056726
rs376641875
rs1003322
rs9398196
rs117512405
rs73581419
rs8102497
rs56061761
rs75664430
rs11584821
rs6669563
rs11769630
rs1611236
rs61405042
rs76666449
rs142730696
rs12451892
rs2763979
rs78491606
rs9600019
rs6054257
rs7772289
rs11412296
rs6881568
rs139795227
rs7099229
rs141214782
rs28502153
rs11991877
rs35446936
rs4724
rs28363070
rs2967355
rs11699829
rs12932179
rs10845387
rs6536702
rs4743037
rs59409453
rs117034449
rs5742915
rs8105767
rs35640778
rs16978028
rs3785074
rs7705526
rs144204502
rs143190905
rs41304832
rs76219171
rs111950327
rs6776756
rs117630647
rs182059586
rs142426306
rs1957937
rs10905255
rs17803849
rs611646
rs6790988
rs17445108
rs4731541
rs11117354
rs9955360
rs10112752
rs79977579
rs1332941
rs80116508
rs932002
rs3891167
rs4530278
rs4498805
rs150150565
rs7790856
rs10773176
rs139669835
rs137901416
rs10768683
rs9940099
rs145114957
rs45604339
rs73730598
rs6590343
rs112394943
rs1291143
rs1985369
rs762679
rs117407747
rs2276182
rs11557154
rs11212631
rs12925933
rs116863223
rs56799554
rs34550383
rs10840270
rs871134
rs76065543
rs939916
rs17677991
rs4616688
rs1907702
rs61748181
rs202034370
rs12412214
rs188918174
rs41269079
rs1023767
rs869785
rs450962
rs185174247
rs3093888
rs2282764
rs7164950
rs13230646
All − MR Egger
All − Inverse variance weighted
−2
−1
0
1
2
MR effect size for
'telomere length || id:ieu−b−4879' on 'IDP_dMRI_ProbtrackX_ISOVF_str_l || id:u
